# PReS13-SPK-1322: Autoinflammatory in nature: what patients teach us

**DOI:** 10.1186/1546-0096-11-S2-I14

**Published:** 2013-12-05

**Authors:** M Gattorno

**Affiliations:** 1 G Gaslini Institute, Genoa, Italy

## 

The autoinflammatory syndromes are a group of multisystem disorders characterized by recurrent episodes of fever and systemic inflammation affecting the eyes, joints, skin, and serosal surfaces. These syndromes differ from autoimmune diseases by several features, including the periodicity whereas autoimmune diseases are progressive, and the lack of signs of involvement of adaptive immunity such as association with HLA aplotypes, high-titer autoantibodies or antigen-specific T cells. Thus, autoinflammatory syndromes are recognized as disorders of innate immunity. This definition is supported by the a dramatic therapeutic response to IL-1 blocking. Indeed, the rapid and sustained response to a reduction in IL-1 activity on an "ex adjuvantibus" basis is the best hallmark of most of these diseases. Due to the rarity of these conditions, most of the studies aimed to unravel the pathogenic consequences related to the mutation of genes involved in inherited autoinflammatory diseases were based on the analysis of in vitro transfected cells or animal models. These approaches has the clear advantage to facilitate the availability of material for these studies and also to reduce the variability associated to clinical and genetic variables (type of mutation, active versus inactive disease, ongoing treatment, etc..). On the other hand the use of patients' primary cells strongly increase the possibility that the observed phenomena could be indeed pertinent to the pathogenesis of the disease and not influenced by possible artifacts linked to the study of transfected cells or animal models.

In the present lecture we will review the contribution that the study of primary cells from patients affected by inherited autoinflammatory diseases gave to the understanding of the role of IL-1 in the pathogenesis of these disorders.

## Disclosure of interest

M. Gattorno Grant/Research Support from: Novartis, Consultant for: Novartis, SOBI, Speakers Bureau: Novartis, SOBI.

